# Analyses of Food-Consumption Data and Migration for the Safety Evaluation of Recycled Polystyrene Intended for Food-Packaging Applications

**DOI:** 10.3390/polym17131846

**Published:** 2025-06-30

**Authors:** David Mittermayr, Wolfgang Roland, Jörg Fischer

**Affiliations:** 1 Institute of Polymeric Materials and Testing, Johannes Kepler University Linz, Altenbergerstrasse 69, 4040 Linz, Austria; joerg.fischer@jku.at; 2 Next Generation Recyclingmaschinen GmbH, Gewerbepark 22, 4101 Feldkirchen an der Donau, Austria; wolfgang.roland@ngr-world.com

**Keywords:** recycling, post-consumer, polystyrene, food-contact application, food consumption, migration, decontamination efficiency

## Abstract

The recycling of post-consumer plastics for food-contact applications is subject to stringent regulatory requirements, particularly with regard to the removal of potentially harmful non-intentionally added substances (NIAS). While polyethylene terephthalate (PET) recycling processes are already approved by the European Food Safety Authority (EFSA), there is a lack of guidance for other polymers like polystyrene (PS). This study aims to provide a scientific basis for assessing the decontamination efficiency required for recycled post-consumer PS in food-contact applications. As one of the first studies to propose a framework for PS decontamination assessment based on EFSA food-consumption data and conservative diffusion modeling, it contributes to filling this regulatory gap. First, European food-consumption data were analyzed to identify critical scenarios of the age-group-dependent intake of PS-packaged food. Based on this, a conservative migration model was applied using a one-dimensional diffusion simulation to determine the maximum allowable initial concentrations of NIAS in PS. The calculated values were then compared with published reference contamination levels to calculate the required cleaning efficiency. The combination of food-consumption values and the migration process showed that trays for fruits and vegetables are the most critical food-contact application for post-consumer PS recycling. The most stringent assumptions resulted in necessary decontamination efficiencies ranging from 92% for the smallest molecule, toluene (92.14 g/mol), to 42% for the largest molecule, methyl stearate (298.50 g/mol). The results provide a methodological basis for regulatory assessments and offer practical guidance for designing safe recycling processes, thereby supporting the circular use of PS in food packaging and building the basis for future regulatory assessments of other polymers, in line with the European Union Plastics Strategy and circular economy objectives.

## 1. Introduction

The global production of plastics has increased exponentially in recent decades. According to PlasticsEurope, over 400 million tons of plastics were produced worldwide in 2023—a large proportion of which was used for packaging, particularly in the food sector [1]. Plastics such as polyethylene (PE), polypropylene (PP), polyethylene terephthalate (PET), and polystyrene (PS) are key materials of modern packaging solutions due to their versatility, ease of processing, and barrier and protective properties [2]. At the same time, the leakage of plastics into the environment and the high persistence of these materials represent a growing environmental concern.

In particular, disposable packaging is a major contributor to plastic waste. Due to its short life, the volume of waste is very high. The European Union has therefore formulated ambitious targets as part of its plastics strategy and the Circular Economy Action Plan: all plastic packaging should be either reusable or recyclable by 2030 [3]. Furthermore, until 2040 the recycled content recovered from post-consumer plastic waste needs to be 25% for contact-sensitive packaging made from plastic materials other than PET, except single-use plastic beverage bottles. In order to achieve these goals, the establishment of new and the expansion of existing closed-loop recycling processes are crucial—especially in the area of food-contact packaging, where the highest standards of hygiene and safety apply [4].

The recycling of post-consumer plastics intended to come into contact with food is subject to strict legal requirements in Europe [3]. The European Food Safety Authority (EFSA) performs a safety assessment of recycling processes for their suitability for sufficiently removing contaminants—in particular so-called non-intentionally added substances (NIAS). A conservative model is generally used here, which assumes that plastics could also have been misused in the consumer cycle. A recycling process is only considered safe if it proves that it can reduce potentially harmful substances below a defined exposure level [5]. Although the EFSA has approved some recycling processes for PET, there are still no clearly defined and standardized evaluation criteria for other plastics, including polystyrene [6]. This is solely due to a lack of data. PS is often used for dairy products like yogurt cups, meat packaging, trays, and other food packaging. However, the recycling rate of PS is low, and the existing applications for recycled PS (rPS) are limited to non-food related areas. Nevertheless, technological advances in sorting, cleaning, and processing are opening up new opportunities to use PS in high-quality, food-safe applications in the future [4]. A key barrier to using post-consumer rPS in food-contact applications is the absence of a scientific framework to determine the required decontamination efficiency for super-cleaning processes (i.e., technology designed to produce food-grade recycled plastics by significantly reducing or eliminating contaminants from post-consumer plastic waste). Unlike PET, for which standardized evaluation procedures and extensive NIAS screening protocols—typically involving comprehensive chemical analyses to identify unknown contaminants—are available, no such systematic approach exists for PS [5,7]. In addition, packaging forms, including storage conditions and material properties such as the diffusion coefficient, processing temperatures, and polymer structure, vary significantly. A key distinction lies in the morphological differences between the polymers: PET is typically semi-crystalline, while PS is largely amorphous. These structural differences strongly influence diffusion behavior, as crystalline regions act as barriers to molecular mobility, leading to lower diffusion rates compared to the more permeable amorphous structures. As a result, the published criteria for PET recycling—such as maximum residual contamination and required cleaning efficiency—cannot be directly transferred and must be re-evaluated for other polymers like PS [4].

**Figure 1:**
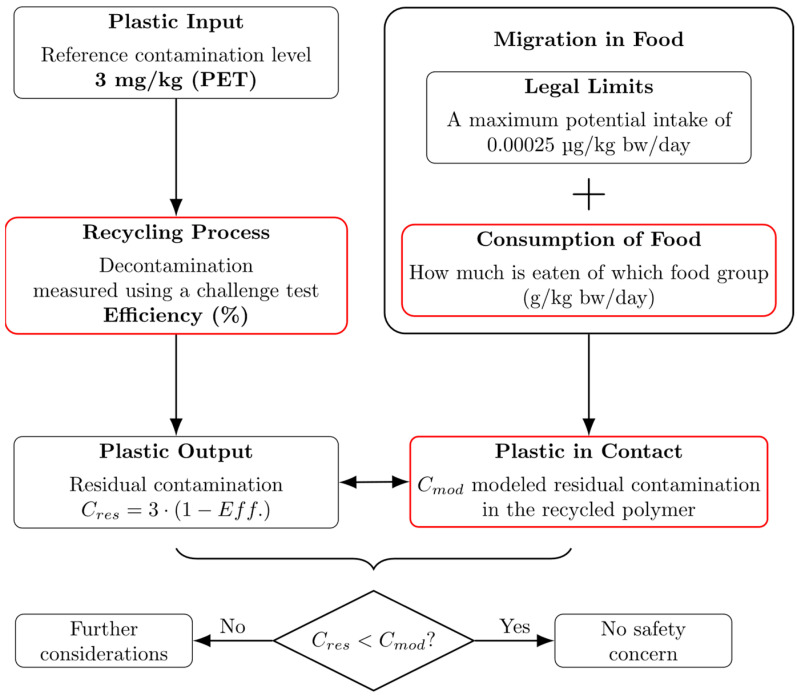
Food-contact safety assessment of PET. Comparison between contamination level of plastic packaging and legal limits of contamination [5]. Investigated and calculated topics of this work are marked with red boxes.

This is the context for the beginning of the present study. The aim is to provide a scientifically based assessment of the minimum decontamination efficiency of the super-cleaning process for the safe use of post-consumer rPS in food-contact applications, similar to the procedure shown for PET in Figure 1. In order to achieve this objective, an analysis was conducted of the consumption data of European consumers with regard to food packaged in PS. The data analysis was based on the publicly available EFSA Comprehensive European Food Consumption Databases [8], whereby different age classes were taken into account and statistically evaluated. Critical conditions for migration were defined from the identified high-consumption scenarios. On this basis, a one-dimensional diffusion simulation was carried out to calculate the migration of hypothetical NIAS from the packaging material into the food. This makes it possible to derive the maximum allowed residual contamination in the polymeric material without exceeding the exposure limits. Finally, by comparing the results with published reference contamination levels (*C_ref_*), the necessary cleaning performance could be determined. There are different approaches for defining *C_ref_*. Due to the limited data available and the fact that there is only one recently published study, there is and can be no fixed assumption for *C_ref_*. Therefore, this paper provides results for both an assumption based on *C_ref_* of PET and an assumption based on recently published information.

The results of this study contribute to the development of an assessment framework for the mechanical recycling of post-consumer polystyrene intended to be used for food-contact packaging applications.

## 2. Methods

In this section, first the statistical calculations for deriving the food-consumption scenarios are shown. The derived scenarios serve as a basis for modeling the migration of potential organic contaminations from the packaging wall into the packed food.

### 2.1. Food-Consumption Data

This study focuses on calculations for food groups which are possibly packed in polystyrene; the picked food categories were yogurt, meat, fish, cheese, fruits, vegetables, and drinks [9]. Furthermore, these categories were combined based on similar packaging forms and storage conditions, as shown in Table 1.

Storage condition A originates out of common storage conditions for the respective food groups [9]. Storage condition B is derived from the testing conditions for migration testing published by the European Union in Regulation No 10/2011 [10]. These standardized testing conditions were selected with respect to the intended food-contact conditions and are presented in Table 1 as storage condition B; however, cold- and hot-filled drinks are exceptions. Drinking cups are not intended for long-term storage. Therefore, the worst foreseeable condition should be applied, which was achieved by using the same values as in storage condition A.

The analyses of this study are based on the food consumption derived by the EFSA Comprehensive Food Consumption Database [8] for the scenarios from Table 1 as well as the migration modeling under the specified storage conditions. The database provides different levels of food groups, starting with a general food group such as “meat and meat products” at the first level and then subdividing it step by step at each level, e.g., meat and meat products subdivided into animal kidney, marinated meat, sausages, etc. The data availability is limited and does not include in-depth background survey data. The survey information is given by country, year, age group, survey name, and food group. The survey results are given in terms of number of consumers, proportion of consumers (%), mean, standard deviation, median, and various percentiles.

The following sections provide the necessary information on the data used, how to statistically calculate the consumption of the combined food groups, and the results of these calculations.

#### 2.1.1. Data Used for Calculations

This study focuses only on the food-consumption data of consumers (non-consumers are excluded) in terms of food consumed per body weight per day (g/kg per day). The inclusion of non-consumers would shift the results towards lower consumption levels. Ignoring the influence of non-consumers provides a worst-case scenario and hence a safe assumption when using the derived data for a safety evaluation. For the calculations, it is necessary to define which data of the database were used, and this information is included in the following chapters.

Available countries

A maximum of 28 countries participated in the surveys and they are included in the EFSA Comprehensive Food Consumption Database. Depending on the food group, consumption data might be missing for several countries. This is due to the fact that information for a particular food group may not be available because it has not been included in every survey. This study considers the available food-consumption data of all European Union members as well as the United Kingdom [8].

Chosen surveys

For many countries, surveys from different years are available. In this study, only the most recent survey (which also met all of the other requirements) was included in the evaluation. This is because older values may be outdated and are no longer relevant.

Chosen population group

Because all analyses are made in relation to the consumer’s body weight, age and size have a major influence on the results. It is therefore necessary to differentiate and focus on the critical population group. Out of the 10 available population groups, the most critical consumption values are given for infants, toddlers, and other children. Additionally, the population group of adults was added for comparison.

Infants (0 to <12 months) may have the highest specific consumption of certain food groups in some surveys and countries, but the significance of this is questionable because their diet is still very one-sided and, in a lot of cases, is heavily dependent on breast milk. Information on meat, fish, and similar foods may not be meaningful. The consumption of fruit and vegetables is generally higher, but mostly in processed form (purees). In addition to polystyrene packaging, purees are most often sold in another form (glass jars), and many parents also make these purees from organic and often home-grown fruit and vegetables.

Toddlers (1 to <3 years) generally have one of the highest food consumptions per body weight because they eat a lot relative to their weight. And from this age onwards, consumption changes to a wider range of foods and there is data available from more surveys than for infants [11]. Therefore, toddlers may be seen as a critical group of consumers for this study.

Other children (3 to <10 years) may also have significant intakes of each food group, but most probably at lower levels than toddlers or infants. This group was included, to account for the fact that children often change their eating habits at a young age.

Adults (18 to <65 years) and all other population groups consume the most food in absolute numbers, but relative to their body weight, they consume less than younger population groups. Therefore, all other groups are not critical for the calculations, but for reference adults were included in this study.

#### 2.1.2. Food-Consumption Data

The mean value of consumption would be the simplest number to calculate for combined food groups, but this number would only be safe for 50% of consumers. A safer number needs to be chosen and therefore the threshold was set at the 95th percentile. This may give a possible overestimate because in combined groups a very high meat consumer will not be the highest fish and cheese consumer. However, it is a safe estimate and has been used in previous calculations of consumption values to be able to calculate migration limits into different food groups [12,13,14,15].

Some groups do not need a statistical combination, such as yogurt (cold- or hot-filled), as the 95th percentile is given directly from the EFSA Comprehensive Food Consumption Database. Other groups require calculation. Some assumptions have to be made in order to statistically evaluate and combine the limited data available. The final 95th percentile values for each country of the defined/combined food group need to be merged to produce a number that reflects each country. It would be possible to again calculate a distribution of the 95th percentile values of the different countries and use this distribution to calculate some sort of cut-off value. This would be an overestimation of the already overestimated numbers and would perhaps be far from realistic consumption values. Therefore, the average of the 95th percentile values was used to obtain a single number representing all countries in the database.

Assumptions and boundaries for statistical calculations

- Survey values are ideally normally distributed. The data for the mean and the standard deviation of the survey values from the EFSA Comprehensive Food Consumption Database can be used for further calculations. Outliers and non-symmetries in the distribution are neglected;
- The consumption patterns of different food groups are theoretically independent of each other. It is assumed that a high consumer of one food group does not necessarily have to be a high or low consumer of another group. The dependency is given by the consumption probabilities;
- Only surveys with more than 60 consumers were considered. Below this number, the 95th percentile would not be statistically robust [11];
- All surveys with more than 60 consumers are considered equivalent. Any data of studies with more than this number of consumers are considered equally important and meaningful;
- Surveys are weighted by the probability of consumption. Consumption values of food groups consumed with a high probability need to be weighted more heavily than those of food groups consumed with a lower probability. For example, if 10% consume fish and 80% consume meat, the mean value and standard deviation of meat consumption are more relevant (the presented values are fictitious percentages for an illustrative purpose only);
- The combination of two food groups consists of three types of consumers: consumers of the first food group only, consumers of the second food group only, and consumers of both food groups. This can be extended in the same sense for the combination of 3 food groups, which gives seven types of consumers;
- The standard deviation or variance for consumers of two or more food groups is weighted by the number of consumers, as standard deviations are highly dependent on the number of consumers.

Calculation of combined food groups

With the calculation method presented in Equation (1), up to three food groups can be combined. An extension of the formula would be required to combine four or more food groups, which is not the case for the defined grouping for polystyrene packaging. The combined mean *µ* solely depends on the probabilities of consumption and the mean values. A simple sample calculation can be found in the Supplementary Materials.

(1) $$\mu =\frac{1}{p_{x}}\left(\sum_{i=1}^{3}\left(p_{i}-\sum_{j\neq i}p_{ij}+p_{123}\right)\mu_{i}+\sum_{1\leq i<j\leq 3}\left(p_{ij}-p_{123}\right)\left(\mu_{i}+\mu_{j}\right)+p_{123}\left(\mu_{1}+\mu_{2}+\mu_{3}\right)\right)$$

**Figure 2:**
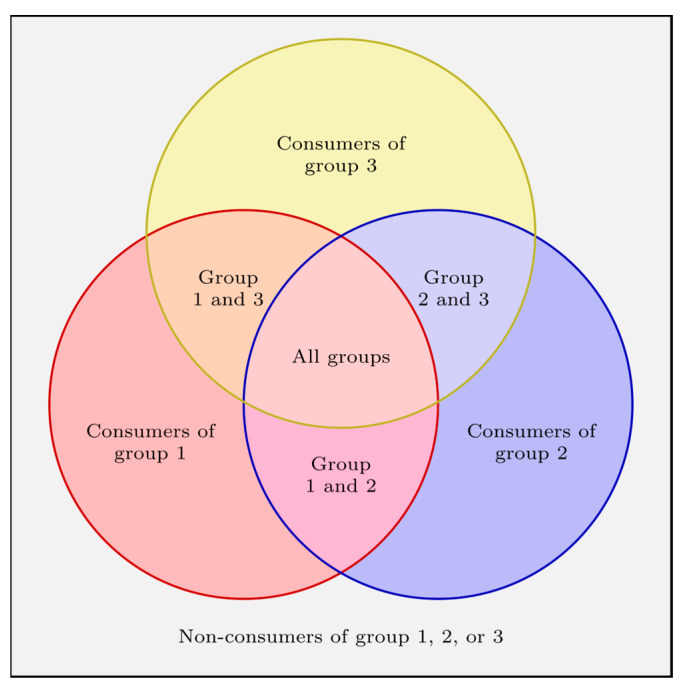
Venn diagram of consumers of three independent food groups. The overlapping areas indicate the concurrent consumption of more than one food group.

The equation contains probabilities *p* and mean consumption data *µ* with indices describing which group these values originate from. Thus, $p_{i}$ stands for the probability of consumption of group i, $p_{ij}$ for the probability of a combined group i and j, and $p_{ijk}$ for the probability of a combined group i, j, and k. This notation has been applied analogously to the mean values μ. $p_{x}$ stands for the probability that a consumer belongs to at least one of the seven consumer groups (illustrated in Figure 2; one of the seven areas of the three overlapping circles); therefore it corrects the calculation to pure consumer values and can be calculated with Equation (2). This correction again leads to higher consumption values, which provide safer estimates for a migration limit calculation. All equations can also be applied for combinations of only two food groups. To do so, the probability of consuming the third food group is zero.

(2) $$p_{x}=\sum_{i=1}^{3}p_{i}-\sum_{1\leq i<j\leq 3}p_{i}p_{j}+p_{1}p_{2}p_{3}$$

The probability that consumers will be in two or three food groups can be calculated with the rule for independent events by multiplication of the individual consumption probabilities of the food groups shown in Equations (3) and (4).

(3) $$p_{ij}=p_{i}p_{j}\;\;\;\;\mathrm{where}\;\;\;1\leq i<j\leq 3$$

(4) $$p_{123}=p_{1}p_{2}p_{3}$$

The square of the standard deviation (variance) of the combined food groups can be calculated using Equation (5).

(5) $$\sigma^{2}=\frac{1}{p_{x}}\left(\sum_{i=1}^{3}p_{i}\left(1-\sum_{j\neq i}p_{ij}+p_{123}\right)\left(\sigma_{i}^{2}+{\mu_{i}}^{2}\right)+\sum_{1\leq i<j\leq 3}p_{i}p_{j}\left(1-p_{k}\right)\left(\sigma_{ij}^{2}+{\left(\mu_{i}+\mu_{j}\right)}^{2}\right)+p_{1}p_{2}p_{3}\left(\sigma_{123}^{2}+{\left(\mu_{1}+\mu_{2}+\mu_{3}\right)}^{2}\right)\right)$$

The unknown parameters in Equation (5) are the standard deviation of consumers of more than one food group. These parameters can be calculated using Equations (6) and (7).

(6) $$\sigma_{ij}^{2}=\frac{n_{i}}{n_{i}+n_{j}}\sigma_{i}^{2}+\frac{n_{j}}{n_{i}+n_{j}}\sigma_{j}^{2}\;\;\;\mathrm{where}\;\;\;1\leq i<j\leq 3$$

(7) $$\sigma_{123}^{2}=\sum_{i=1}^{3}\frac{n_{i}}{N}\sigma_{i}^{2}$$

where N stands for the sum of the consumer numbers of group 1 ($n_{1}$), group 2 ($n_{2}$), and group 3 ($n_{3}$), shown in Equation (8).

(8) $$N=n_{1}+n_{2}+n_{3}$$

Using the mean value and standard deviation of the combined food group, the 95th percentile ($x_{95}$) of the ideally normally distributed consumption function can be calculated using Equation (9).

(9) $$x_{95}=\mu +1.65\sigma$$

### 2.2. Migration Modeling

**Figure 3:**
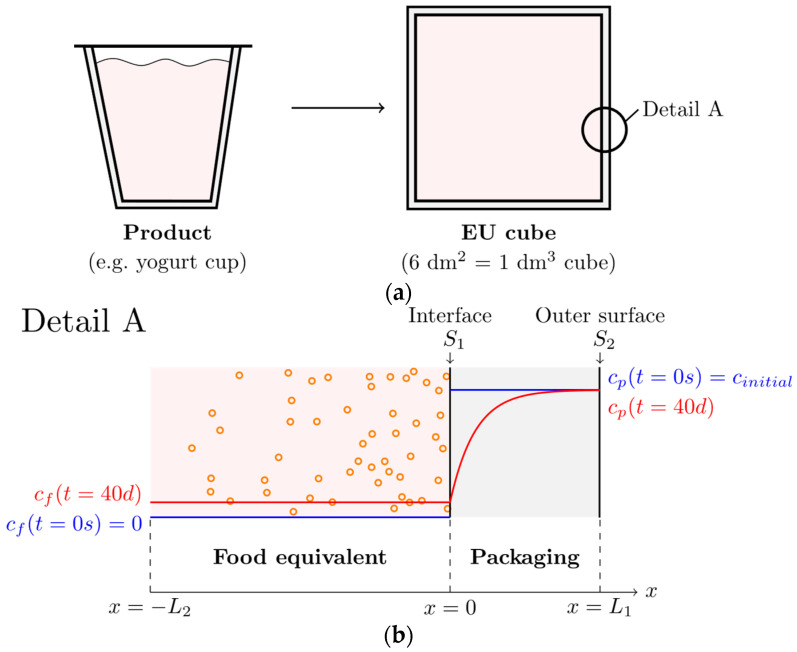
Migration modeling. (**a**) Transfer of product geometry to the EU cube model with subsequent diffusion process. (**b**) Detail A: schematic representation of the migration of contaminants at interface S_1_ from the packaging to the food equivalent. The blue line shows the initial conditions/concentrations, and the red line shows the final concentration distribution of the contamination for the food equivalent (pink) and the packaging (grey).

The next step after calculating the food-consumption values was to determine the migration of residual contaminations from the packaging into the food, given through the defined storage conditions (Table 1). First, a defined volume to surface ratio was given by the EU cube model, where a cube filled with 1 dm^3^ of food equivalent and a packaging contact surface of 6 dm^2^ simplifies any packaging geometry in a representative way, as shown in Figure 3a [10]. The chosen packaging thickness of L_1_ = 0.3 mm completes the geometry, which may overestimate most packaging geometries, but which is a conservative assumption.

It is assumed that the food equivalent has no contamination level, and the packaging has an evenly distributed contamination level at the time the food is filled into its packaging. During storage, the contamination level in the food equivalent is increasing while the residual contaminations migrate from the packaging wall into the food, resulting in a reduction of the contamination level in the packaging. This migration is a diffusion-driven process, and hence a gradual concentration profile will be formed in the packaging wall as shown in Figure 3b. To predict this curve, a diffusion model for estimating specific migration was used to investigate the diffusion coefficient, which can be calculated using Equations (10) and (11) with the information given in Table 2 [16].

(10) $${A_{P}}^{*}={A_{P}}^{\prime *}-\frac{\tau }{T}$$

(11) $$D_{P}^{*}=D_{0}\exp\left({A_{P}}^{*}-c_{1}M_{r}^{\frac{2}{3}}+c_{2}M_{r}-\frac{c_{3}}{T}\right)\;\;\;\mathrm{where}\;\;\;D_{0}=1\;cm²/s$$

${D_{P}}^{*}$ gives an upper-bound estimate of the diffusion coefficient and is therefore a safe assumption to calculate with. Migration-model parameters for high-impact polystyrene (HIPS) were chosen because the higher polymer specific parameter ${A_{P}}^{\prime *}$ leads to faster diffusion processes and is therefore also a safe assumption due to the worst-case scenario. The molecular weight range for the calculation was set from 92 g/mol to 430 g/mol in order to include all proposed surrogate substances suggested for the challenge test of PET recycling processes [5].

Analytical solutions exist for this type of diffusion problem, but they are limited to single scenario, monolayer problems [17]. A numerical solution was therefore required, which would be already available in commercial software such as SML Software (AKTS SA, Sierre, Switzerland). Due to the limited availability of free software, and in order to make each parameter setting possible, a custom solution was created.

An iterative model was programmed in Python 3.13 (Python Software Foundation, Fredericksburg, VA, USA) based on Fick’s second law of diffusion given in Equation (12) for one-dimensional problems [18]. As the analytical solution is also based on this diffusion model, and due to its versatility, simplicity, and applicability, it was chosen for this study. Fick’s second law describes the rate of change of concentration *c* with respect to the rate of change of time *t*, which equals a diffusion coefficient times the second spatial derivative of the concentration with respect to position *x*.

(12) $$\frac{\partial c}{\partial t}=D\;\frac{\partial^{2}c}{\partial x^{2}}$$

Two boundary conditions are required to solve the diffusion equation. The first boundary was the Neumann condition on the outer surface (S_2_ in Figure 3b) of the packaging where air contact would occur, which gives a constant gradient at one position. This gradient was set to zero, so therefore there was no material transport on the outer surface of the packaging.

As the second boundary, the Dirichlet boundary condition was chosen at the interface (S_1_ in Figure 3b) between the packaging and the food equivalent. The condition states that the concentration at the surface is constant and equal to the concentration in the food equivalent. For each time-iteration, the interface concentration was constant for the dimensional iteration over the packaging thickness starting with a value of zero (equal to the concentration of the food equivalent before the diffusion process). These two boundary conditions are safety assumptions: perfect isolation on the outer surface and perfect molecular transition on the inner surface. They have been shown to be applicable for such problems [17,19,20]. In addition, a stirred-food equivalent (uniformly distributed concentrations in the medium) had to be assumed, which also resulted in a slight overestimation of migration.

From the first spatial derivative of the concentration profile at the interface (S_1_), the diffusive flux of the surrogates from the packaging into the food equivalent was determined. This flux information, combined with the contact area and volume of the food equivalent, allowed the calculation of the increase in average concentration within the food equivalent over time. To describe the increase in concentration in the food equivalent $\frac{dc_{\mathrm{food}}}{dt}$, the rate of change is linked to the diffusion-driven mass flow at the interface ${\left.\frac{\partial c}{\partial x}\right|}_{x=0}$ shown in Equation (13). Where *A* is the contact area between the packaging and the food equivalent, $\rho_{\mathrm{food}}$ is the food equivalent density, $V_{\mathrm{food}}$ is the volume of the food equivalent, and $\rho_{packaging}$ is the density of the packaging.

(13) $$\frac{dc_{\mathrm{food}}}{dt}=\frac{A}{\rho_{\mathrm{food}}\cdot V_{\mathrm{food}}}\cdot D\cdot \rho_{packaging}\cdot {\left.\frac{\partial c}{\partial x}\right|}_{x=x_{\mathrm{Interface}}}$$

For this study, a discretization of 40,000 elements over the thickness of the packaging and a time iteration step size of 1000 s was investigated. The discretization was checked with the Courant–Friedrichs–Lewy condition and was adjusted by varying the time step size Δt until the stability parameter α was 0.5, keeping the spatial step size Δx constant, shown in Equation (14) [21]. Finer discretization over the thickness had no effect on the results.

(14) $$\alpha =\frac{D\cdot \Delta t}{\Delta x^{2}}\leq \frac{1}{2}$$

**Figure 4:**
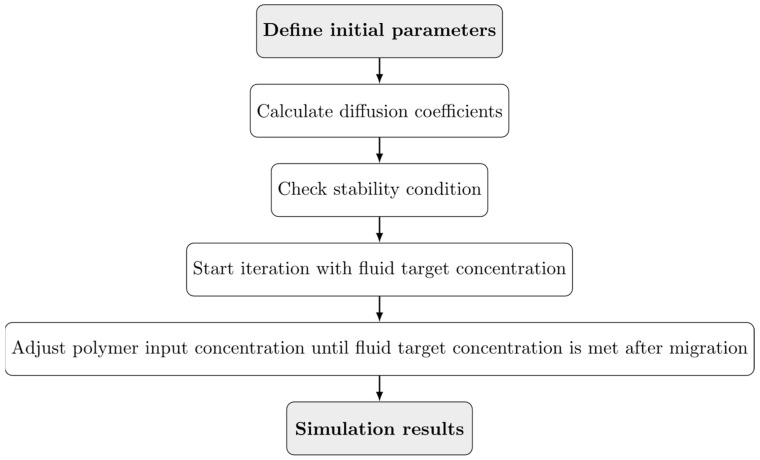
Flowchart of the migration simulation with a loop condition in the penultimate step.

EFSA has recognized the conservative nature of this diffusion prediction model for PET and has proposed a general overestimation factor (OEF) of 5 for molecules below 150 Da and an OEF of 10 above 150 Da [5,22]. However, these default OEFs only marginally reflect the true extent of migration, especially for low diffusivity polymers such as PET and PS. For low-volatility substances, the OEF may exceed 250. Although OEFs tend to decrease with increasing temperatures, they remain well above 5 even at 40 °C [9]. In reality, higher temperatures are only reached for short periods and are not responsible for the major migration. To be conservative and comparable with the EFSA approach for PET, an OEF of 5 was applied to the migration simulation for molecules below 150 Da and an OEF of 10 for molecules above 150 Da. A schematical flowchart of migration simulation is illustrated in Figure 4. The simulations were calculated using information on the maximum potential intake per day and per body weight of 0.0025 µg of non-intentionally added substances, the polystyrene density of 1.04 g/cm^3^, a food equivalent density of 1.00 g/cm^3^, the storage conditions, and the consumption values.

The final results of the migration modeling give numbers for the allowed contamination in the packaging *C_mod_* to comply with legal limits. The necessary decontamination efficiency can be calculated with *C_ref_* of post-consumer polystyrene, shown in Equation (15).

(15) $$Dec.\;Eff.=1-\frac{C_{mod}}{C_{ref}}$$

A conservative assumption about the contamination of post-consumer PS would be to estimate the same level of contamination as for PET, as they both have a low diffusivity. PET is more often misused for the storage of harmful liquids than PS, simply because of the closed geometry of bottles compared to cups and trays. Studies on the contamination level of PET have led to a *C_ref_* of 3 ppm by EFSA [5]. A recently published article investigated the contamination level for PS. Sampling over 3 years and 6 countries suggests that the *C_ref_* of PS is 1 ppm including safety factors [15]. In order not to claim the validity of one *C_ref_* or the other, both scenarios were calculated.

## 3. Results and Discussion

The results section is divided into two parts. The first part presents the food-consumption results. This information is then used as input for the migration simulation—the results of which are presented in the second part of this section.

### 3.1. Food-Consumption Results

This section contains consumption values for the defined six food groups for the critical population groups of infants, toddlers, and other children. For comparison, the consumption values of adults are also included.

First, the consumption values of each group were calculated without combining any food groups. It was found that toddlers ate at least twice as much of the selected food categories compared to adults, as shown in Table 3. These values were all taken from the EFSA Comprehensive Food Consumption Database.

There are also other ways to define the consumption values, such as recommended values for water or critical consumption values for defined ages. An example would be the highest consumption of fluids by infants in the first 16 weeks of life, where its recommended that they should consume up to 260 mL/(kg × day) [14,23]. At this age, infants are expected to be fed, exclusively breastfed and/or formula-fed. Infant formula, especially the water for it, can be either out of glass bottles, PET bottles, or from the tap, but not out of a polystyrene packaging and this can therefore be neglected. In 2016, EFSA published general food-consumption data for a risk-assessment guideline for chemicals in food, which states four categories with a stepwise condition check. The first category is based on the recommended fluid intake for infants mentioned above. The second category gives a consumption value for liquids such as milk, milk products, and other non-alcoholic drinks of 80 g/(kg × day), which is very similar to the consumption values in Table 3 for the category of water and water-based beverages [24]. Due to the more detailed listing of the second category with better classification of different storage conditions of the packaged food products, the calculated consumption values were used.

The combined food-group consumption values are shown in Table 4. The first food group, yogurt, the fourth group, hot-filled drinks, and the fifth group, cold-filled drinks, did not require a combination of food groups, e.g., because fermented milk and cream products can be assumed to be all yogurts for safety reasons.

The other entries in Table 4 are combined values and show the food consumption of four population groups, according to which infants and toddlers reach the highest and most critical values. Therefore, other children and adults do not need to be considered when talking about critical population groups for the consumption of polystyrene-packed goods. Infants’ consumption of fruits and vegetables is similar to that of toddlers, but their consumption of yogurt, meat, fish, and cheese are different.

The difference between the first, fourth, and fifth rows of values in Table 3 and Table 4 shows the imperfections of the survey values and the assumed ideal normal distribution. These imperfections display the need to increase the calculated numbers by 11% in order to account for the largest offset deviation between the calculated and the given values. This deviation was found between water and water-based beverages and cold-filled drinks for the infant population group. The final numbers have been rounded up to whole numbers and are shown in Table 5.

### 3.2. Migration Modeling Results

For the purposes of this section, only the highest, most critical consumption values were chosen for the simulation of the six different food groups with defined storage conditions. The calculations were performed for eight selected substances (toluene, chlorobenzene, chloroform, methyl salicylate, phenylcyclohexane, benzophenone, lindane, and methyl stearate), which are the surrogate contaminants proposed for the challenge test of PET recycling processes [5].

**Figure 5:**
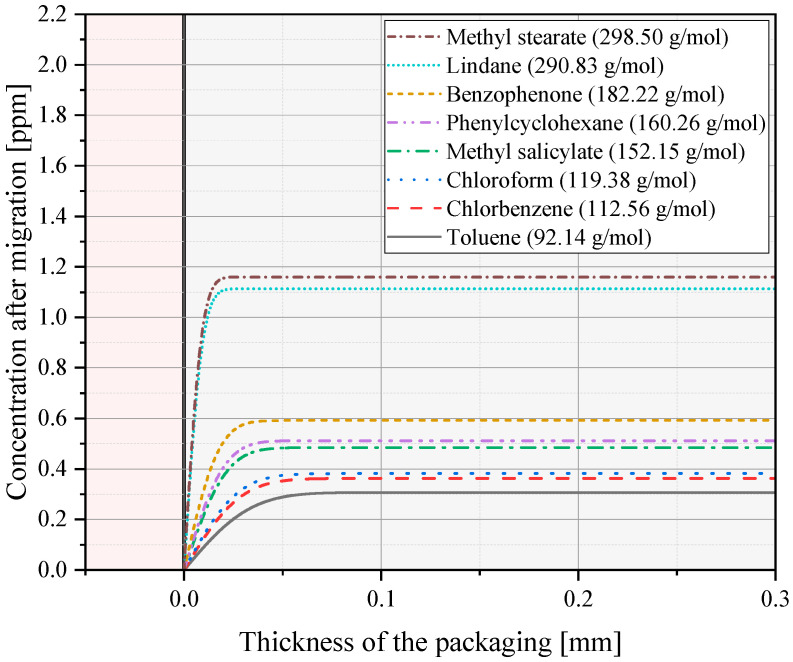
Concentration curves for different molecules after 30 days at 25 °C (trays for the food group fruits and vegetables with storage condition A) with an OEF of 5. The grey colored part shows the 0.3 mm thick packaging and the pink part illustrates the food equivalent with final concentrations in the parts per billion (ppb) region.

The results for the fruit and vegetables food group trays with storage condition A are shown in Figure 5. The results show that a packaging thickness of 0.1 mm would give the same results as there is no change in concentration visible after 0.1 mm of packaging. As no changes are shown for positions further away from the food equivalent, the maximum residual contamination levels at the start of migration are indicated by horizontal lines. Exact figures are given in Table 6. The table shows that larger molecules with higher molecular weight show a slower migration behavior than smaller molecules due to their inhibited movement through the polymer matrix, and therefore higher concentrations are allowed in the packaging material.

**Figure 6:**
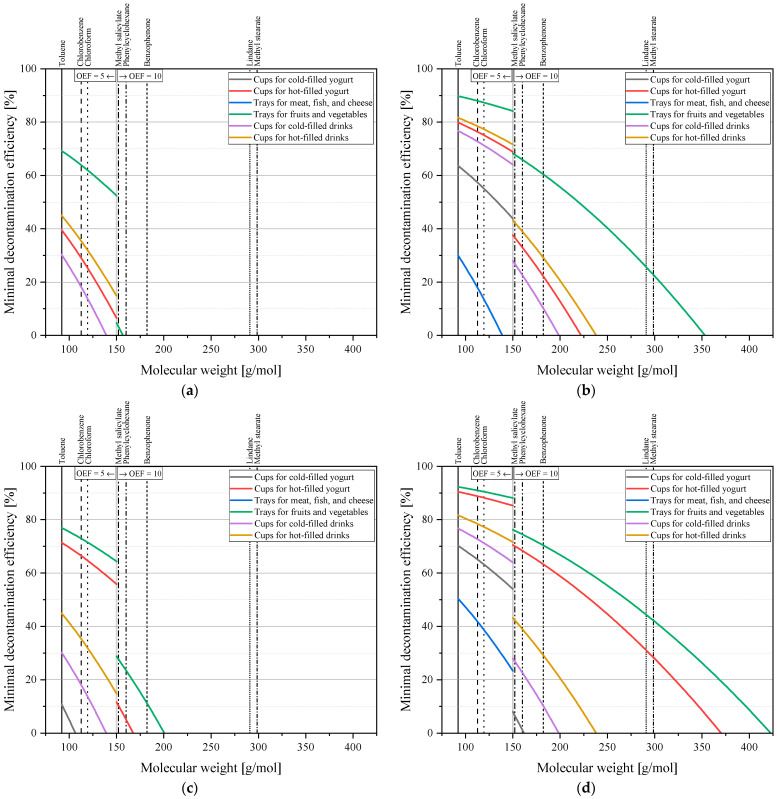
Necessary decontamination efficiencies for a safe post-consumer polystyrene recycling process for storage condition A for a *C_ref_* of (**a**) 1 ppm and (**b**) 3 ppm and for storage condition B for the same *C_ref_* of (**c**) 1 ppm and (**d**) 3 ppm. The grey vertical line shows the molecular weight at which the OEF changed from 5 to 10.

For storage conditions A and B and both scenarios of *C_ref_* (1 ppm and 3 ppm) for post-consumer PS, the necessary decontamination efficiencies for a safe polystyrene recycling process are shown in Table 7 for the eight surrogate contaminants. These results are also shown in Figure 6, each with a quadratic fit over the values of the eight molecules and a coefficient of determination R^2^ of 0.9999.

As of now, EFSA has not provided guidelines for rPS for food-contact applications, so technology developers are currently left with two options: either wait for an official position or proceed by selecting one of the four provided storage condition and *C_ref_* combinations. Once a scenario is chosen, Table 7 provides the corresponding minimum decontamination efficiencies for the eight surrogate contaminants. These values must be met or exceeded to qualify the PS recycling process for food-contact applications. For example, if a developer selects storage condition A with a *C_ref_* of 1 ppm and intends to cover all food-packaging applications, the minimum required decontamination efficiency for toluene is 70%. If this level (or the levels for any other surrogate) cannot be achieved, the application scope must be narrowed—e.g., to applications with lower necessary decontamination efficiencies or to applications with age-based restrictions.

The ranking of the different molecules is of course the same for both *C_ref_* on each set of storage conditions. The worst-case application is trays for fruits and vegetables, showing the highest required decontamination efficiencies for storage conditions A and B. For the 3 ppm assumption of *C_ref_* (3 ppm), approximately 90% decontamination efficiency is required for small molecules such as toluene and approximately 23% decontamination efficiency is required for larger molecules such as methyl stearate for storage condition A. Storage condition B is more critical than the storage condition A. The most critical values are for the worst-case application (trays for fruits and vegetables) above 92% for the smallest molecule (toluene) and above 42% for the biggest molecule (methyl stearate). This scenario obtained the highest values because of high-consumption values paired with warm and long storage conditions. Besides the values for drinks, where their storage conditions were set equally, storage condition B required overall higher decontamination efficiencies. This difference is due to the different defined storage conditions and it shows that the temperature has a significantly greater influence on the diffusion process than time. Increasing the temperature by a factor of 1.6 has a greater impact on the level of contamination than increasing the time by a factor of three.

For a *C_ref_* of 1 ppm, the curves lay much lower. Starting at roughly 70% necessary decontamination efficiency for toluene, and with a limit of around 156 g/mol, above this, no more cleaning is required for the worst-case application for storage condition A. Storage condition B with a *C_ref_* of 1 ppm requires around 77% decontamination efficiency for toluene and also shows a limiting molecular weight where no more cleaning is necessary at approximately 201 g/mol. Such limits are due to the slower migration process of bigger molecules, which fall below EFSA’s exposure threshold even without any decontamination.

Looking at other storage scenarios, cold-filled yogurt and trays for meat, fish, and cheese, the results showed the lowest necessary cleaning efficiencies, with almost no decontamination required for the molecular weight range obtained for *C_ref_* of 1 ppm. Yogurt cups, which are the most common packaging made out of PS, are the second or third most critical application when being hot-filled. The necessary decontamination efficiencies for toluene are 40% (*C_ref_* of 1 ppm) and 80% (*C_ref_* of 3 ppm) for storage condition A and 72% (*C_ref_* of 1 ppm) and 91% (*C_ref_* of 3 ppm) for storage condition B. The molecular weight limits above decontamination are no longer necessary at 150 g/mol (*C_ref_* of 1 ppm) and 222 g/mol (*C_ref_* of 3 ppm) for storage condition A and at 200 g/mol (*C_ref_* of 1 ppm) and 370 g/mol (*C_ref_* of 3 ppm) for storage condition B.

Moreover, real-world food packaging systems show considerable variability in several dimensions: (1) packaging geometry, such as the use of trays, pouches, or rigid containers, which affect surface contact and exposure; (2) food type, where solids generally lead to lower migration than aqueous or fatty foods due to reduced partitioning and slower diffusion; and (3) consumer behavior, including storage time and temperature, which often deviate from worst-case assumptions used in modeling. For example, fruits and vegetables typically have limited contact areas with the packaging, they are more likely to be solid rather than liquid, and they are often peeled, which removes the highest contaminated part. They often come from different sources and come in different or non-packed forms (home-grown). These factors collectively indicate that the actual migration levels are likely lower than those predicted in this study. As such, the calculated concentrations and required decontamination efficiencies represent a conservative upper boundary designed to ensure safety under the most protective assumptions.

The findings of this study provide a robust, data-driven foundation that can be used for various purposes when it comes to advancing the use of rPS for food-contact applications. First, the results provide EFSA with a scientifically robust basis on which to develop future guidelines for the safety evaluation of rPS. Second, the approach yields concrete exposure-based decontamination targets derived from worst-case consumption and migration scenarios. These targets allow recycling-technology developers to align their process design with regulatory safety requirements and prepare evaluation dossiers accordingly. Third, the methodology linking age-specific dietary exposure with diffusion modeling is not limited to polystyrene. It can be adapted to other polymers and can thus serve as a blueprint for the structured assessment and future approval of additional recycled plastics for use in contact with food.

## 4. Conclusions

This study provides a scientific basis for assessing the safety of recycled polystyrene (rPS) intended to be used for food-contact applications from post-consumer sources by analyzing consumption data from the Comprehensive European Food Consumption Database from the European Food and Safety Authority (EFSA) and combining them with statistical methods to calculate the consumption values for defined food groups. The necessary calculations were based on ideal normally distributed consumption data weighted by the probability of consumption. Infants and toddlers were identified as the most critical population groups due to their higher intake relative to body weight. Adults, on the other hand, do not even have half of the specific consumption that infants or toddlers do. The calculated consumption values were used to model worst-case migration scenarios for different food groups and storage conditions using a one-dimensional diffusion approach. Migration simulations with eight representative surrogates revealed that permissible initial concentrations in rPS packaging depend strongly on the molecular characteristics of the substances. The results showed a clear dependence on molecular size: smaller, more mobile molecules require stricter limits, while larger molecules can be tolerated at higher initial concentrations due to slower diffusion through the polymer matrix. Based on these migration limits and published contamination data, the required decontamination efficiencies were calculated for two scenarios: a recently published reference contamination level (*C_ref_)* of 1 ppm and a *C_ref_* of poly-ethylene terephthalate of 3 ppm. The results of the calculated decontamination efficiencies give the necessary cleaning performance required to meet food-safety standards.

Standardized storage conditions for migration testing (storage condition B) revealed higher necessary decontamination efficiencies than common storage conditions for foods packed in rPS (storage condition A). The worst-case application for rPS is trays for fruit and vegetables for both chosen storage conditions. The combination of relatively high temperatures (25 °C or 40 °C) and long storage conditions (30 days or 10 days), together with high consumption levels, makes the application most critical for the migration of contaminants. Necessary decontamination efficiencies ranged between 70% and 92% for the smallest calculated molecule toluene (92.14 g/mol) and for the biggest calculated molecule methyl stearate (298.50 g/mol) between 0% and 42%. As larger molecules migrate more slowly, most scenarios showed a limit molecular weight. Above this limit, no decontamination is needed to achieve the given legal exposure thresholds. Yogurt cups are the most common type of polystyrene food-contact packaging. Hot-filled yogurt cups needed the second or third highest cleaning efficiencies. The required decontamination efficiencies range up to approximately 40% to 91% for toluene and between 0% and 28% for methyl stearate.

Unlike mechanical recycling for PET, there are currently no guidelines for the mechanical recycling of PS. Ultimately, EFSA will need to define which scenarios of input contamination levels and storage conditions must be applied for the safety assessment of rPS. As of now, there is not enough information available to support this decision, and it is expected that EFSA will gather relevant data through monitoring reports submitted by technology developers working on novel mechanical recycling processes for PS. The results presented in this publication cover a range of possible reference contamination levels and migration conditions, providing a scientific basis for assessing the required decontamination efficiency of a super-cleaning process for rPS intended for food-contact applications. In doing so, this study (1) supports EFSA in establishing future guidelines for food-grade rPS, (2) provides concrete numerical targets that recycling technology developers can use to design and validate their processes, and (3) introduces a transferable methodology that can serve as a blueprint for the safety evaluation and approval of other recycled polymers for food-contact applications.

## Figures and Tables

**Table 1:** Food groups combined based on similar packaging/packaging conditions displayed with their common storage condition (storage condition A) and standardized testing condition for migration testing (storage condition B) [9,10].

Food Group	Storage Condition A	Storage Condition B
Cold-filled yogurt	40 days at 6 °C	10 days at 20 °C
Hot-filled yogurt	2 h at 70 °C followed by 40 days at 6 °C	10 days at 40 °C
Meat, fish, and cheese	30 days at 6 °C	10 days at 20 °C
Fruits and vegetables	30 days at 25 °C	10 days at 40 °C
Cold-filled drinks	1 day at 25 °C	1 day at 25 °C *
Hot-filled drinks	2 h at 70 °C	2 h at 70 °C *

* No long-term storage. Already the worst foreseeable condition copied from storage condition A.

**Table 2:** Diffusion-model parameters given for PS and high-impact polystyrene with an upper temperature limit and a relative molecular weight range where the model has been validated [16].

Polymer	Temperature	Molecular Weight	Polymer Specific Parameters		Constants		
	T [K]	$M_{r}$ [g/mol]	${A_{P}}^{\prime *}$ [-]	τ [K]	$c_{1}$ [(g/mol)^−2/3^]	$c_{2}$ [(g/mol)^−1^]	$c_{3}$ [K]
Polystyrene	≤343.15	104–647	−1.0	0	0.1351	0.003	10,454
High-impact polystyrene	≤343.15	104–430	1.0	0			

**Table 3:** Food consumption in grams per kilogram of body weight per day of consumers. Numbers only for the population groups infants, toddlers, other children, and adults. Food groups chosen according to the categories in Table 1.

Food Category	Infants	Toddler	Other Children	Adult
	[g/(kg × day)]	[g/(kg × day)]	[g/(kg × day)]	[g/(kg × day)]
Fermented milk or cream	24.61	18.74	11.33	3.71
Meat and meat products	8.75	10.06	8.24	4.44
Fish (meat)	4.97	5.72	4.46	2.43
Cheese	8.86	6.47	3.79	1.74
Fruit and fruit products	23.63	23.61	15.74	6.17
Vegetables and vegetable products	16.31	18.33	13.60	5.72
Water and water-based beverages	74.22	72.18	52.60	31.03
Hot drinks and similar (coffee, cocoa, tea, and herbal infusions)	29.67 *	26.40	13.29	14.17

* Data originated from only 3 surveys.

**Table 4:** Calculated combined food consumption in grams per kilogram of body weight per day of consumers. Numbers only for the population groups infants, toddlers, other children, and adults.

Food Category	Infants	Toddler	Other Children	Adult
	[g/(kg × day)]	[g/(kg × day)]	[g/(kg × day)]	[g/(kg × day)]
Cold-filled yogurt	22.30	17.36	10.53	3.45
Hot-filled yogurt	22.30	17.36	10.53	3.45
Meat, fish, and cheese	9.11	12.73	9.22	4.85
Fruits and vegetables	27.16	26.70	17.83	7.71
Cold-filled drinks	66.83	67.43	49.94	29.06
Hot-filled drinks	29.44	25.55	12.46	13.31

**Table 5:** Corrected combined food consumption in grams per kilogram of body weight per day of consumers. Numbers only for the population groups infants, toddlers, other children, and adults. Numbers were corrected by an imperfection-factor of 11% and were rounded to integers.

Food Category	Infants	Toddler	Other Children	Adult
	[g/(kg × day)]	[g/(kg × day)]	[g/(kg × day)]	[g/(kg × day)]
Cold-filled yogurt	25	20	12	4
Hot-filled yogurt	25	20	12	4
Meat, fish, and cheese	11	15	11	6
Fruits and vegetables	31	30	20	9
Cold-filled drinks	75	75	56	33
Hot-filled drinks	33	29	14	15

**Table 6:** The calculated maximum input contamination levels of the PS packaging applications not exceeding legal limits for eight surrogate contaminants given in parts per million (ppm).

	Food-Group Packaging Application	Toluene	Chloro- benzene	Chloro- form	Methyl Salicylate	Phenyl- cyclohexane	Benzo- phenone	Lindane	Methyl Stearate
		92.14 g/mol	112.56 g/mol	119.38 g/mol	152.15 g/mol	160.26 g/mol	182.22 g/mol	290.83 g/mol	298.50 g/mol
Storage condition A	Cups for cold-filled yogurt	1.09	1.28	1.35	3.43	3.63	4.20	7.91	8.23
	Cups for hot-filled yogurt	0.60	0.71	0.75	1.90	2.01	2.33	4.38	4.56
	Trays for meat, fish, and cheese	2.09	2.47	2.60	6.61	6.99	8.08	15.22	15.83
	Trays for fruits and vegetables	0.31	0.36	0.38	0.97	1.02	1.18	2.23	2.32
	Cups for cold-filled drinks	0.70	0.82	0.87	2.20	2.32	2.69	5.07	5.28
	Cups for hot-filled drinks	0.55	0.65	0.68	1.73	1.83	2.12	3.99	4.15
Storage condition B	Cups for cold-filled yogurt	0.89	1.05	1.11	2.81	2.97	3.43	6.46	6.73
	Cups for hot-filled yogurt	0.28	0.34	0.35	0.90	0.95	1.10	2.07	2.15
	Trays for meat, fish, and cheese	1.48	1.75	1.84	4.68	4.95	5.72	10.77	11.21
	Trays for fruits and vegetables	0.23	0.27	0.29	0.72	0.77	0.89	1.67	1.73
	Cups for cold-filled drinks	0.70	0.82	0.87	2.20	2.32	2.69	5.07	5.28
	Cups for hot-filled drinks	0.55	0.65	0.68	1.73	1.83	2.12	3.99	4.15

**Table 7:** Necessary decontamination efficiencies for *C_ref_* of 1 ppm and 3 ppm of the eight surrogate contaminants for storage conditions A and B. Values for molecules below 150 Da calculated with an OEF of 5 and above 150 Da with an OEF of 10.

	*C_ref_*	Application	Toluene	Chloro- Benzene	Chloro- Form	Methyl Salicylate	Phenyl- Cyclohexane	Benzo- Phenone	Lindane	Methyl Stearate
			92.14 g/mol	112.56 g/mol	119.38 g/mol	152.15 g/mol	160.26 g/mol	182.22 g/mol	290.83 g/mol	298.50 g/mol
Storage condition A	1 ppm	Cups for cold-filled yogurt	0.00%	0.00%	0.00%	0.00%	0.00%	0.00%	0.00%	0.00%
		Cups for hot-filled yogurt	39.75%	28.86%	25.00%	0.00%	0.00%	0.00%	0.00%	0.00%
		Trays for meat, fish, and cheese	0.00%	0.00%	0.00%	0.00%	0.00%	0.00%	0.00%	0.00%
		Trays for fruits and vegetables	69.32%	63.78%	61.82%	3.11%	0.00%	0.00%	0.00%	0.00%
		Cups for cold-filled drinks	30.50%	17.91%	13.46%	0.00%	0.00%	0.00%	0.00%	0.00%
		Cups for hot-filled drinks	45.14%	35.22%	31.71%	0.00%	0.00%	0.00%	0.00%	0.00%
	3 ppm	Cups for cold-filled yogurt	63.79%	57.24%	54.92%	0.00%	0.00%	0.00%	0.00%	0.00%
		Cups for hot-filled yogurt	79.92%	76.29%	75.00%	36.55%	32.91%	22.42%	0.00%	0.00%
		Trays for meat, fish, and cheese	30.30%	17.69%	13.23%	0.00%	0.00%	0.00%	0.00%	0.00%
		Trays for fruits and vegetables	89.77%	87.93%	87.27%	67.70%	65.85%	60.52%	25.76%	22.69%
		Cups for cold-filled drinks	76.83%	72.64%	71.15%	26.74%	22.53%	10.38%	0.00%	0.00%
		Cups for hot-filled drinks	81.71%	78.41%	77.24%	42.22%	38.91%	29.35%	0.00%	0.00%
Storage condition B	1 ppm	Cups for cold-filled yogurt	11.16%	0.00%	0.00%	0.00%	0.00%	0.00%	0.00%	0.00%
		Cups for hot-filled yogurt	71.54%	66.41%	64.59%	10.13%	4.99%	0.00%	0.00%	0.00%
		Trays for meat, fish, and cheese	0.00%	0.00%	0.00%	0.00%	0.00%	0.00%	0.00%	0.00%
		Trays for fruits and vegetables	77.05%	72.91%	71.44%	27.53%	23.38%	11.41%	0.00%	0.00%
		Cups for cold-filled drinks	30.50%	17.91%	13.46%	0.00%	0.00%	0.00%	0.00%	0.00%
		Cups for hot-filled drinks	45.14%	35.22%	31.71%	0.00%	0.00%	0.00%	0.00%	0.00%
	3 ppm	Cups for cold-filled yogurt	70.39%	65.03%	63.14%	6.42%	1.06%	0.00%	0.00%	0.00%
		Cups for hot-filled yogurt	90.51%	88.80%	88.20%	70.04%	68.33%	63.38%	31.16%	28.32%
		Trays for meat, fish, and cheese	50.65%	41.72%	38.56%	0.00%	0.00%	0.00%	0.00%	0.00%
		Trays for fruits and vegetables	92.35%	90.97%	90.48%	75.84%	74.46%	70.47%	44.49%	42.20%
		Cups for cold-filled drinks	76.83%	72.64%	71.15%	26.74%	22.53%	10.38%	0.00%	0.00%
		Cups for hot-filled drinks	81.71%	78.41%	77.24%	42.22%	38.91%	29.35%	0.00%	0.00%

## Data Availability

The raw data supporting the conclusions of this article will be made available by the authors on request.

## Supplementary Materials

The following supporting information can be downloaded at: https://www.mdpi.com/article/10.3390/polym17131846/s1, Sample Calculation: Combination of two food groups.

## Abbreviations

The following abbreviations are used in this manuscript:

*C_ref_*	Reference contamination level
EFSA	European Food and Safety Authority
HIPS	High impact polystyrene
NIAS	Non-intentionally added substances
PE	Polyethylene
PET	Polyethylene terephthalate
PP	Polypropylene
PS	Polystyrene
ppm	Parts per million
ppb	Parts per billion
OEF	Over estimation factor
rPS	Recycled polystyrene
