# Supplementary material for: Analyses of Food-Consumption Data and Migration for the Safety Evaluation of Recycled Polystyrene Intended for Food-Packaging Applications
Source: Polymers (Basel). 2025 Jun 30;17(13):1846. doi: 10.3390/polym17131846 (PMC12251960; doi:10.3390/polym17131846)
Supplement: Supplementary file 1 [file polymers-17-01846-s001.zip › polymers-3707652-supplementary.pdf]

Supplementary Materials

Sample calculation for the combination of the food groups meat and fish. For simplicity and claritiy only two food groups get combined. The values of the third group are set to zero, leading to a possible neglection of some terms in the calculation.

Table S1. Values originated from the EFSA Comprehensive European Food Consumption Databases showing the 2014 survey EU Menu Austria: Food consumption data for Austrian adults.

Food Groups	Mean Consumption [g/(kg·day)]	Standard Deviation of Consumption Values [g/(kg·day)]	Probability of Consumption [%]	Consumer Numbers [-]
Meat	$\mu_M = 1.85$	$\sigma_M = 1.37$	$p_M = 86.8$	$n_M = 1{,}883$
Fish	$\mu_F = 0.88$	$\sigma_F = 0.69$	$p_F = 28.6$	$n_F = 621$
None	$\mu_0 = 0$	$\sigma_0 = 0$	$p_0 = 0$	$n_0 = 0$

The Equations (S1) to (S9) from the main text are repeated here:

$$\mu = \frac{1}{p_x}\left(\sum_{i=1}^{3}\left(p_i - \sum_{j\neq i} p_{ij} + p_{123}\right)\mu_i + \sum_{1\leq i<j\leq 3}(p_{ij} - p_{123})(\mu_i + \mu_j) + p_{123}(\mu_1 + \mu_2 + \mu_3)\right) \quad \text{(S1)}$$

$$p_x = \sum_{i=1}^{3} p_i - \sum_{1\leq i<j\leq 3} p_i p_j + p_1 p_2 p_3 \quad \text{(S2)}$$

$$p_{ij} = p_i p_j \quad where \quad 1 \leq i < j \leq 3 \quad \text{(S3)}$$

$$p_{123} = p_1 p_2 p_3 \quad \text{(S4)}$$

$$\sigma^2 = \frac{1}{p_x}\left(\sum_{i=1}^{3} p_i\left(1 - \sum_{j\neq i} p_{ij} + p_{123}\right)(\sigma_i^2 + {\mu_i}^2) + \sum_{1\leq i<j\leq 3} p_i p_j (1 - p_k)\left(\sigma_{ij}^2 + \left(\mu_i + \mu_j\right)^2\right) + p_1 p_2 p_3(\sigma_{123}^2 + (\mu_1 + \mu_2 + \mu_3)^2)\right) \quad \text{(S5)}$$

$$\sigma_{ij}^2 = \frac{n_i}{n_i + n_j}\sigma_i^2 + \frac{n_j}{n_i + n_j}\sigma_j^2 \quad where \quad 1 \leq i < j \leq 3 \quad \text{(S6)}$$

$$\sigma_{123}^2 = \sum_{i=1}^{3}\frac{n_i}{N}\sigma_i^2 \quad \text{(S7)}$$

$$N = n_1 + n_2 + n_3 \quad \text{(S8)}$$

$$x_{95} = \mu + 1.65\sigma \quad \text{(S9)}$$

Equations (S1) to (S9) are reordered, simplified, and reformulated for the given food groups. Each variable is calculated using exact numbers and rounded to a reasonable number of digits.

$$N = n_M + n_F = 2{,}504 \quad \text{(S8)}$$

$$\sigma_{123}^2 = \frac{n_M}{N}\sigma_M^2 + \frac{n_F}{N}\sigma_F^2 = 1.53 \quad \text{(S7)}$$

$$\sigma_{MF}^2 = \frac{n_M}{n_M + n_F}\sigma_M^2 + \frac{n_F}{n_F + n_M}\sigma_F^2 = 1.53 \quad \text{(S6)}$$

$$p_{123} = p_M p_F p_0 = 0 \quad \text{(S4)}$$

$$p_{MF} = p_M p_F = 24.8, \qquad p_{M0} = 0, \qquad p_{F0} = 0 \tag{S3}$$

$$p_x = (p_M + p_F) - (p_M p_F) = 0.91 \tag{S2}$$

$$\mu = \frac{1}{p_x}(((p_M - p_{MF})\mu_M + (p_F - p_{MF})\mu_F) + (p_{MF}(\mu_M + \mu_F))) = 2.05 \tag{S1}$$

$$\sigma^2 = \frac{1}{p_x}(p_M - p_{MF})(\sigma_M^2 + {\mu_M}^2) + (p_F - p_{MF})(\sigma_F^2 + {\mu_F}^2) + p_M p_F(\sigma_{MF}^2 + (\mu_F + \mu_M)^2) = 1.39 \tag{S5}$$

$$x_{95} = \mu + 1.65\sigma = 4.34 \tag{S9}$$

The combined food group of meat and fish from the given survey has an mean value of 2.05 g/(kg·day), a standard deviation of 1.39 g/(kg·day), a 95th percentile of 4.34 g/(kg·day), and a probability of consumption of 91 %.
